# Supplementary material for: A Systematic Review of the Reliability and Validity of the Patient Activation Measure Tool
Source: Healthcare (Basel). 2024 May 24;12(11):1079. doi: 10.3390/healthcare12111079 (PMC11171848; doi:10.3390/healthcare12111079)
Supplement: Supplementary file 1 [file healthcare-12-01079-s001.zip › Table S1.pdf]

Supplementary Material

Table S1. Full search strategy for the various databases

Medline Search

Patient Activation	("patient activation" or PAM).ti,ab.
Healthcare outcomes or engagement or adherence	"quality of health care"/ or clinical competence/ or guideline adherence/ or "outcome and process assessment (health care)"/ or "clinical care".ti,ab. or exp "Delivery of Health Care"/ or attitude to health/ or "Continuity of Patient Care"/ or Patient-Centered Care/ or patient engagement/ or patient participation/ or health knowledge, attitudes, practice/ or health education/ or "goal setting".ti,ab. or "coordination of care".ti,ab.

Embase Search

Patient Activation	'patient activation':ti,ab OR pam:ti,ab
Healthcare outcomes or engagement or adherence	'health care quality'/exp OR 'clinical competence'/exp OR 'protocol compliance'/exp OR 'treatment outcome'/exp OR 'clinical care':ti,ab OR 'health care delivery'/exp OR 'attitude to health'/exp OR 'patient care'/exp OR 'patient engagement'/exp OR 'patient participation'/exp OR 'goal setting':ti,ab OR 'coordination of care':ti,ab
	'article'/it

Cochrane

#1	(("patient activation" or PAM)):ti,ab,kw	S	1368
#2	MeSH descriptor: [Quality of Health Care] explode all trees	MeSH	603527
#3	MeSH descriptor: [Clinical Competence] explode all trees	MeSH	4385
#4	MeSH descriptor: [Guideline Adherence] explode all trees	MeSH	1383
#5	MeSH descriptor: [Outcome Assessment, Health Care] explode all trees	MeSH	191967
#6	MeSH descriptor: [Attitude to Health] explode all trees	MeSH	45858
#7	MeSH descriptor: [Continuity of Patient Care] explode all trees	MeSH	36255
#8	MeSH descriptor: [Patient-Centered Care] explode all trees	MeSH	1045
#9	MeSH descriptor: [Patient Participation] explode all trees	MeSH	2067
#10	MeSH descriptor: [Health Knowledge, Attitudes, Practice] explode all trees	MeSH	7406
#11	MeSH descriptor: [Health Educators] in all MeSH products	MeSH	32
#12	("goal setting" or "coordination of care"):ti,ab,kw	S	2983
#13	#2 or #3 or #4 or #5 or #6 or #7 or #8 or #9 or #10 or #11 or #12		622291
#14	#1 and #13		520
